# Prognostic Value of Interleukin-32 Expression and Its Correlation with the Infiltration of Natural Killer Cells in Cutaneous Melanoma

**DOI:** 10.3390/jcm10204691

**Published:** 2021-10-13

**Authors:** Ji Young Kang, Kyung Eun Kim

**Affiliations:** 1 Department of Health Industry, Sookmyung Women’s University, Seoul 04310, Korea; ellykang@sookmyung.ac.kr; 2 Department of Cosmetic Sciences, Sookmyung Women’s University, Seoul 04310, Korea

**Keywords:** interleukin-32 (IL-32), cutaneous melanoma, multiomic analysis, immune cell infiltration, natural killer (NK) cells

## Abstract

Interleukin-32 (IL-32) is well known as a proinflammatory cytokine that is expressed in various immune cells and cancers. However, the clinical relevance of IL-32 expression in cutaneous melanoma has not been comprehensively studied. Here, we identified the prognostic value of *IL32* expression using various systematic multiomic analyses. The *IL32* expressions were significantly higher in cutaneous melanoma than in normal tissue, and Kaplan–Meier survival analysis showed a correlation between *IL32* expression and good prognosis in cutaneous melanoma patients. In addition, we analyzed the correlation between *IL32* expression and the infiltration of natural killer (NK) cells to identify a relevant mechanism between *IL32* expression and prognosis in cutaneous melanoma (*p* = 0.00031). In the relationship between *IL32* expression and the infiltration of NK cells, a negative correlation was found in resting NK cells (rho = −0.38, *p* = 3.95 × 10^−17^) whereas a strong positive correlation was observed only in active NK cells (rho = 0.374, *p* = 1.23 × 10^−16^). Moreover, *IL32* expression was markedly positively correlated with the cytolytic molecules, such as granzyme and perforin. These data suggest that *IL32* expression may increase patient survival through the infiltration and activation of NK cells, representative anticancer effector cells, in cutaneous melanoma. Collectively, this study provides the prognostic value of *IL32* expression and its potential role as an effective predictive biomarker for NK cell infiltration in cutaneous melanoma.

## 1. Introduction

Despite its relatively low incidence rate, melanoma is the most critical type of cancer due to its high levels of malignancy and invasive activity [1,2]. Cutaneous melanoma, a type of melanoma, is characterized by high metastasis and poor prognosis; therefore, it accounts for 75% of skin-related deaths [3]. In recent decades, the incidence of cutaneous melanoma has continued to increase worldwide, with approximately 96,000 new cases in 2019 [4]. Various methods have been used to treat melanoma, but achieving significant therapeutic effects is difficult due to its high resistance to classical therapies, such as chemotherapy and radiotherapy [5,6,7]. Recently, various immunotherapies, such as vaccines and immuno-checkpoint inhibitors, have attempted to improve the side effects and to overcome resistance. To date, monoclonal antibodies targeting the checkpoint molecules cytotoxic T lymphocyte-associated protein 4 (CTLA-4) and programmed cell death protein 1 (PD-1), and the ligand PD-L1 are well known as the most effective immunotherapies [8,9]. These tumor immunotherapies have been established as key treatments for the clinical treatment of cancer [10]. The composition and immune contexture of the tumor microenvironment (TME) are closely related to the successful response of immunotherapy, and an increase in specific cells in the TME is associated with increased survival of patients with various types of cancer [11,12,13]. Thus, the interaction of melanoma cells with immune cells within the TME significantly influences tumor proliferation, differentiation, and progression [14,15]. Melanoma is a major immunogenic tumor, and the number of related studies using immunotherapy to control immune responses is increasing. In particular, melanoma cells are known to regulate their close association with the TME to promote tumor progression, invasion, and metastasis [16,17]. Composed of a complex network of immune cells, various growth factors, and cytokines, the TME is closely associated with melanoma, which affects the treatment effect [18].

The presence, localization, and phenotype of tumor-infiltrating lymphocytes (TILs) within the TME have been predicted to determine immunotherapy and the key regulators of disease progression [19,20,21]. TILs contain not only effector cells but also immunosuppressive cells such as regulatory T lymphocytes, tumor associated macrophage (TAM), and myeloid-derived suppressor cells (MDSC) as contributors to anticancer effects [22,23]. Therefore, an infiltration of effector cells including natural killer (NK) cells and CD8^+^ T cells in the tumor is highly correlated with a good prognosis in cancer patients [24,25]. The presence and activation of NK cells in the TME is associated with tumor suppression [26,27,28,29]. NK cells, a major effector cell of the innate immune system, are the first line of defense in the anticancer immune system [30]. When NK cells are activated, cytolytic molecules are released to induce apoptosis of tumor cells, and cytokines such as interferon-gamma (IFN-γ) and tumor necrosis factor alpha (TNF-α) are produced to regulate adaptive immune T cell-mediated immune responses [26,27,28,29]. NK cell receptors induced by proinflammatory cytokines are associated with NK cell activity in patients with melanoma and are known to improve cell toxicity in tumors [31]. To predict the prognosis of cutaneous melanoma, understanding the role of TILs in the tumor environment is necessary.

Interleukin-32 (IL-32) was first demonstrated as a natural killer cell transcript (NK4), which was detected in activated NK cells and T cells [32]. IL-32 contains eight small exons and is located on human chromosome16p13.3 [33]. IL-32, a proinflammatory cytokine differentially expressed in IL-18-responsive cells, induces the expressions of TNF-α, IL-1β, and C–X–C chemokine family members and activates the nuclear factor kappa-light-chain-enhancer of activated B cells (NF-κB) and p38 mitogen-activated protein kinase (MAPK) pathways [34,35,36]. IL-32 is more highly expressed in immune cells than in nonimmune cells and is closely related to anticancer effects in various types of cancer [37,38]. IL-32 has been known to affect tumor death by regulating immune cells including NK cells. NK cells release TNF-related apoptosis-inducing ligand (TRAIL), TNF, Fas ligand (FASL), and death receptor 3 (DR3) ligand to induce cancer cell death. Moreover, IL-32 expression increased the number of NK cells and CD8^+^ T cells in blood and recruited NK cells and CD8^+^ T cells in tumor tissues. Recently, a novel role of IL-32 for immunotherapy has been suggested by identifying the mechanism by which IL-32 primes CD8^+^ T cells and recruits activating intratumoral DCs and macrophages [39]. In contrast, several reports show the procancer effects of IL-32. The overexpression of IL-32 increases the tumor size and lymph node metastasis in breast cancer, and IL-32 expression is also associated with tumor metastasis and cancer cell migration in gastric and lung cancers [40,41,42].

As such, while studies with conflicting results have reported contradictory effects of IL-32 in various tumors, a comprehensive analysis of the clinical relevance of *IL32* expression has yet to be performed [37,38,43]. Based on various databases, we investigated the correlation between *IL32* expression levels and cancer patient outcomes. In addition, the correlation between *IL32* expression and NK cells, which are effector cells, was analyzed to confirm the impact on cutaneous melanoma survival rate. The results of this study identified the important role of *IL32* in cutaneous melanoma and provided its interaction and function with TILs.

## 2. Materials and Methods

### 2.1. IL32 mRNA Expression in Various Tumors

Gene Expression Profiling Interactive Analysis (GEPIA2; http://gepia.cancer-pku.cn/, accessed on 21 March 2021) was used to compare the *IL32* mRNA expression between various tumors and their normal tissues. GEPIA2 provides customizable functionalities based on data from The Cancer Genome Atlas (TCGA) and the Genotype Tissue Expression project (GTEx) [44]. GEPIA2 also offers *IL32* mRNA levels in cutaneous melanoma (SKCM; skin cutaneous melanoma) and normal tissues. The transcription of *IL32* expression levels between SKCM and normal tissues was shown with box plots.

### 2.2. Analysis of the Correlation between IL32 Expression and Prognostic Value

To estimate the correlation between *IL32* mRNA expression and patient survival in SKCM, various web tools were used. The prognostic value of *IL32* expression was analyzed in TCGA datasets using the OncoLnc (http://www.oncolnc.org/, accessed on 28 March 2021) database and GEPIA2. We compared the survival of two groups of patients with low and high *IL32* mRNA expression levels and provided hazard ratios (HR) with 95% confidence intervals and Kaplan–Meier (KM) survival curves, and the log-rank *p*-values were analyzed using GEPIA2. We used the TIMER databases to evaluate the clinical relevance of *IL32* expression. TIMER v.1.0 provides KM plots to visualize the survival differences and *p*-values of the log-rank test to compare the survival curves (log-rank *p* < 0.05). HR indicates the hazard ratio, and its lower and upper 95% confidential intervals (CIs) were shown in each plot [45]. TIMER v.2.0 also offers Cox regression results including Z-score and statistical significance *p*-value. A KM plot was performed using a Cox proportional hazard model with clinical factors including race, gender, and tumor stage. Z-scores were compared to assess whether the outcome of the gene expression modulated by clinical factors was significant (increased risk: *p* < 0.05, Z > 0; decreased risk: *p* < 0.05, Z < 0; and not significant: *p* > 0.05) [46]. The Cox regression results of the clinical factors including gender, tumor stage, race, and tumor purity are presented in Supplementary Table S1.

### 2.3. Analysis of IL32 Gene Mutations in Skin Cutaneous Melanoma (SKCM)

A comprehensive open-source platform, cBioportal (http://www.cbioportal.org/, accessed on 2 April 2021) provides various cancer genomic datasets. To investigate the *IL32* gene mutation in SKCM, we used the cBioportal database version 3.2.14, which provides various cancer genomic datasets [47,48]. The mutation diagram of the *IL32* gene was generated using default parameter settings. The statistical analysis was performed using an unpaired *t*-test of GraphPad 7 software. The genomic alterations of *IL32* include copy number amplification (CNA), deletion, and nonsense and missense mutations with unknown significance. In addition, we analyzed the promoter methylation of *IL32* using the UALCAN for the TCGA-SKCM dataset. The statistical method of UALCAN was used to analyze changes in the expression levels between normal and other tumor grades, and *p* < 0.05 was considered statistically significantly different.

### 2.4. Correlation between IL32 Expression and Infiltration of Various Immune Cells

Tumor immune system interactions (TISIDB, http:// cis.hku.hk./TISIDB/index.php, accessed on 25 May 2021) is a database used to analysis the relative abundance of tumor immune systems and TILs. Here, it was used to determine the interactions between *IL32* and TILs in SKCM. TIMER is an online web tool used to systematically analyze the correlation of immune infiltrates with various factors such as gene expression and prognostic values. TIMER v.1.0 was used to analyze the comprehensive correlation between *IL32* mRNA and tumor-infiltrating immune cell signatures. The correlation between *IL32* expression levels and the abundances of immune infiltrates including tumor purity, B cells, CD8^+^ T cells, CD4^+^ T cells, macrophages, neutrophils, and dendritic cells, was visualized using scatter plots. In addition, we analyzed the correlation between *IL32* expression and the gene markers of infiltrating NKs using the GEPIA2. We used TIMER v.2.0 (http://timer.cistrome.org/, accessed on 5 April 2021) to confirm the significance of *IL32* expression with activated NK cells [49]. In addition, we compared the activated NK cell infiltration levels and resting NK cell infiltration levels using CIBERSORT in TIMER v.2.0 to determine the interactions between *IL32* and TILs in SKCM.

### 2.5. Correlation between Coexpressed Genes and IL32

We used the TCGA-SKCM dataset in cBioportal to analyze the coexpression genes of *IL32* expression. Then, we identified the 25 strongest correlated genes with the highest Spearman correlation value and the lowest *p*-value. We used the UCSC Xena browser (https://xena.ucsc.edu/, accessed on 13 May 2021) to analyze the correlation between *IL32* and the gene with the highest positive correlation using a heatmap and scatter plot with TCGA-SKCM. The R2 database was used to visualize the correlation of *IL32* with the highest positive correlation using the Tumor melanoma metastasis Bhardwaj-44 dataset via scatter plot. We also analyzed the correlation between *IL32* and its altered genes to identify gene ontology terms using Enricher (http://amp.pharm.mssm.edu/Enrichr/, accessed on 25 April 2021).

## 3. Results

### 3.1. The Expression Analysis of IL32 in Various Types of Cancers

**Figure 1:**
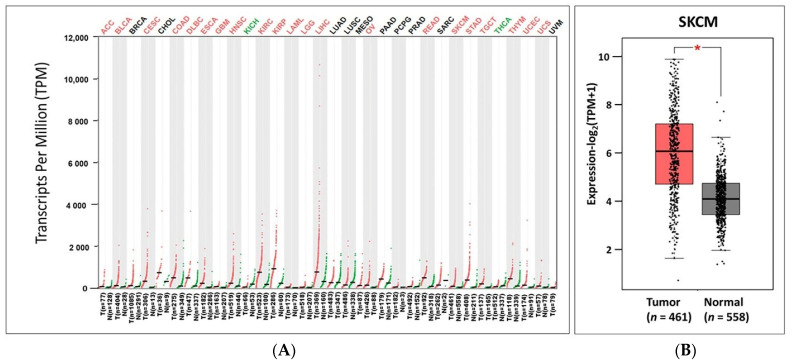
*IL32* mRNA expression levels in various types of cancers and their normal tissues. (**A**) Difference in *IL32* expressions between tumor (red) and normal tissues (green) using the GEPIA2 database. Supplementary Table S2 presents the abbreviations of various types of cancers. (**B**) The box plots represent the *IL32* mRNA expressions in SKCM (*n* = 461) and normal tissues (*n* = 558) using the GEPIA2. * *p* < 0.05.

To analyze *IL32* mRNA expression in tumors and normal tissues, we identified *IL32* mRNA levels using various databases. GEPIA2 showed that the *IL32* mRNA expression levels were significantly elevated in most types of tumors, including cutaneous melanoma (SKCM; skin cutaneous melanoma), as indicated in red (Figure 1A). However, the level of *IL32* mRNA expression was higher in normal tissues than in tumor tissues of kidney chromophobe (KICH) and thyroid carcinoma (THCA), as indicated in green. Detailed findings of particular tumor types are compiled in Supplementary Table S2. We further compared the mRNA levels of *IL32* between SKCM (461 samples) and normal (558 samples) tissues based on data from TCGA and GTEx. Figure 1B shows that the *IL32* expression was significantly higher in SKCM tissues than in normal tissues (*p* < 0.05). Collectively, the data from all databases showed that *IL32* mRNA expression in SKCM was markedly higher than that in normal tissues.

### 3.2. Correlation between IL32 Expression and Patient Survival in Various Types of Cancers

**Figure 2:**
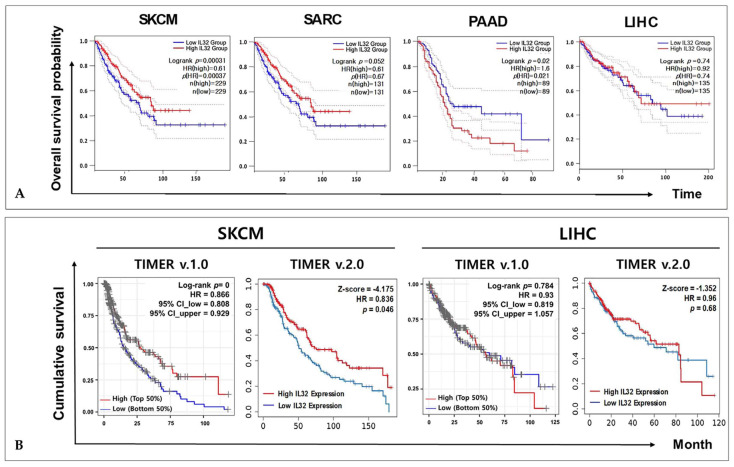
Correlation between *IL32* expression and the prognosis of various types of cancers. Kaplan–Meier survival curves generated using the GEPIA2 website indicate that *IL32* expression is higher (red) and lower (blue) than the median value of the TCGA data (*p* < 0.05): (**A**) SKCM, SARC, PAAD, and LIHC; (**B**) cumulative survival curves demonstrating the survival rates of patients with high (blue) or low (red) *IL32* expression using the TIMER web tools (log-rank *p* < 0.05, Z < 0).

To investigate the correlation between *IL32* mRNA expression and patient survival rate in various types of cancers, the overall survival probabilities were compared using the Cox regression model and the OncoLnc online tool. The Cox regression results for *IL32* mRNA in various cancer types are shown in Supplementary Table S3. A Cox regression analysis for *IL32* expression was performed for four types of cancers: SKCM, sarcoma (SARC), pancreatic adenocarcinoma (PAAD), and liver hepatocellular carcinoma (LIHC) (*p* < 0.01). As shown in Figure 2A, we found that a higher *IL32* expression level was correlated with a better overall survival of SKCM (log-rank *p* = 0.00031) and PAAD (log-rank *p* = 0.02). No significant correlation was found between patient survival in SARC (log-rank *p* = 0.052) and LIHC (log-rank *p* = 0.74). The correlations between *IL32* expression and disease free survival (DFS) of various types of cancers by GEPIA2 are shown in Supplementary Figure S4. Interestingly, the *IL32* mRNA expression in LIHC was significantly elevated, as in SKCM (Figure 1A); therefore, LIHC was used as a control in subsequent analyses. To evaluate the clinical relevance of *IL32* expression, we further identified the cumulative survival of patients with SKCM and LIHC using TIMER web tools. The KM plots were performed on a Cox proportional hazard model to determine the significance of *IL32* expression on outcome, and the Cox regression results including log-rank *p*-value and Z-score were provided. As shown in Figure 2B, a higher *IL32* expression was associated with a better prognosis in SKCM (log-rank *p* = 0, HR = 0.866) in the TIMER v.1.0 database. Moreover, the TIMER v.2.0 database also shows that an *IL32* mRNA expression was associated with good prognosis for SKCM patients (Z-score = –4.175). The Cox regression results adjusted by clinical factors such as race, age, gender, and tumor stages are shown in Supplementary Table S1. In contrast, no significant correlation was found between *IL32* expression and patient survival in LIHC (log-rank *p* = 0.784, HR = 0.93, Z-score = −1.352). These findings suggest that *IL32* mRNA expression influences the prognosis of patients with SKCM.

### 3.3. Genome Change of IL32 Expression in Melanoma

**Figure 3:**
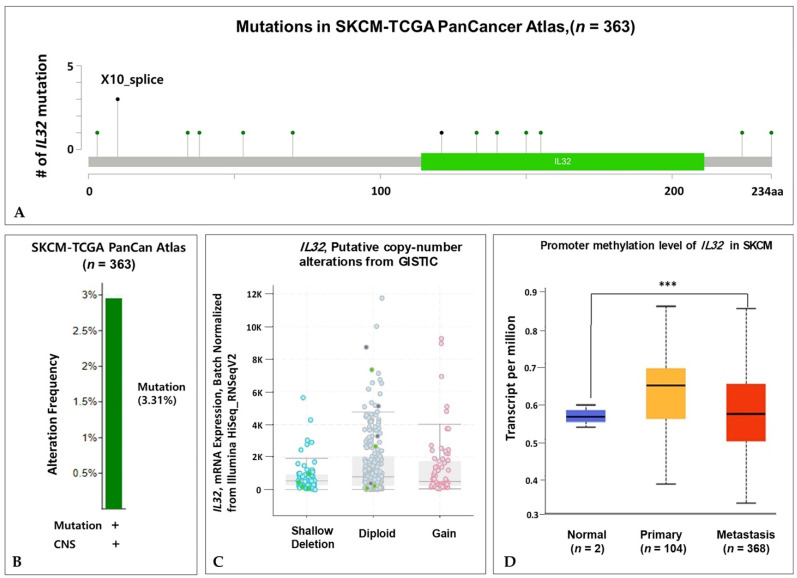
Genome alterations and *IL32* expression in SKCM. (**A**) The lollipop plot shows the type and location of each mutation frequency in the TCGA-SKCM PanCan Atlas dataset using the cBioPortal database (*n* = 363). (**B**) The mutations of the *IL32* gene expression are presented in SKCM. (**C**) The correlation between *IL32* expression and CNA status of shallow deletion (SD), diploid (D), and gain (G). (**D**) The box plots reveal the promoter methylation levels of *IL32* in the TCGA-SKCM dataset using the UALCAN database. Normal vs. Primary (*p* = 4.23 × 10^−1^), Normal vs. Metastasis (*p* = 5.95 × 10^−5^), and Primary vs. Metastasis (*p* = 9.15 × 10^−1^); *** *p* < 0.001.

We explored the status of *IL32* gene alteration in SKCM using cBioportal. A total of 363 patient samples were included from the TCGA database, and mutation types including 11 missense, 3 splice, and 1 nonsense mutation(s) were observed in the *IL32* coding region (Figure 3A). The X10 splice mutation was confirmed three times more often than the other mutations. Fifteen mutations in the *IL32* gene are shown in Supplementary Table S5. Figure 3B shows that the alteration frequency of the *IL32* gene was 3.3% in the TCGA PanCan Atlas dataset. Additionally, we analyzed the correlation between BRAF mutation status and *IL32* expression to identify other mutations in SKCM. BRAF mutations have been observed in several types of cancer, such as melanoma and colorectal cancer, and are associated with cancer cell growth and proliferation [50]. As shown in Supplementary Figure S6, no significant difference was found between mutated BRAF and wild-type BRAF with *IL32* expression in melanoma (*p* = 0.064, *p* < 0.05). To further examine whether the *IL32* CNA status was associated with mRNA expression, we analyzed *IL32* mRNA expression for each CNA status. The *IL32* expression was markedly lower in the shallow deletion samples than in the diploid and gain samples (Figure 3C). However, we found no significant differences in the *IL32* expression between the diploid and gain samples. These data suggest that a shallow deletion of the CNA status could contribute to the high expression of *IL32* in SKCM. To further analyze the methylation status of the *IL32* gene in SKCM, we investigated the TCGA-SKCM dataset using the UALCAN database. As shown in Figure 3D, promoter methylation was significantly increased in metastatic melanoma (*p* = 5.95 × 10^−5^) compared with that in primary tissues (*p* = 4.23 × 10^−1^). Promoter methylation is an epigenetic regulator, and increased methylation is indicative of tumors [51]. Taken together, these results suggest that a positive correlation is found between DNA methylation and the mRNA expression of *IL32*.

### 3.4. Correlation of IL32 Expression with Immune Infiltrates

**Figure 4:**
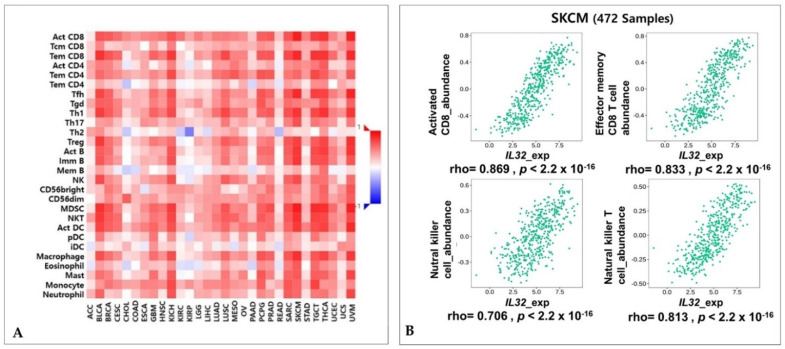
Correlations between *IL32* expression with lymphocytes. (**A**) Correlation between the expressions of *IL32* and lymphocytes in various cancer types. (**B**) The expression of *IL32* was significantly correlated with the immune cells in SKCM: activated CD8^+^ T cell, effector CD8^+^ T cell, natural killer (NK) cell, and NKT T cell (rho > 0.4, *p* < 0.001).

The fact that *IL32* expression is involved in various cancer malignancies, including breast cancer (BRCA) and colon adenocarcinoma (COAD), is well known. However, our data showed that a high *IL32* expression is associated with high survival rates. To determine the mechanism associated with clinical relevance, we investigated the correlation between *IL32* expression levels and immune cell infiltration in SKCM. Supplementary Figure S7 shows that an analysis of the data using TIMER v.1.0 shows that a positive correlation is found between *IL32* expression and immune cell infiltration in SKCM. In contrast, the *IL32* expression levels were not significantly correlated with tumor purity and immune infiltrates in LIHC. These results demonstrate that *IL32* in SKCM may be expressed by infiltrated immune cells. We further identified significant correlations of *IL32* with 28 types of TILs among various cancers using the web portal TISIDB (Figure 4A). In Figure 4A, the *IL32* expression has a positive correlation with various immune cells in many types of cancer. In the correlation between *IL32* and NK cell infiltration, only five types of cancer including SKCM show significant positive correlations (Supplementary Figure S8). *IL32* expression was markedly correlated with the abundance of NK cells (rho = 0.706, *p* < 2.2 × 10^−16^), natural killer T (NKT) cells (rho = 0.813, *p* < 2.2 × 10^−16^), activated CD8^+^ T cells (rho = 0.869, *p* < 2.2 × 10^−16^), and effector memory CD8^+^ T cells in SKCM (rho = 0.833, *p* < 2.2 × 10^−16^) (Figure 4B). Overall, these data suggest that the expression of higher *IL32* is significantly involved in activated NK cell and CD8^+^ T cell infiltration and suggest that this leads to the antitumor activity of the effector cell.

### 3.5. Correlation between IL32 Expression and Various Subsets of Immune Cells in Melanoma

**Figure 5:**
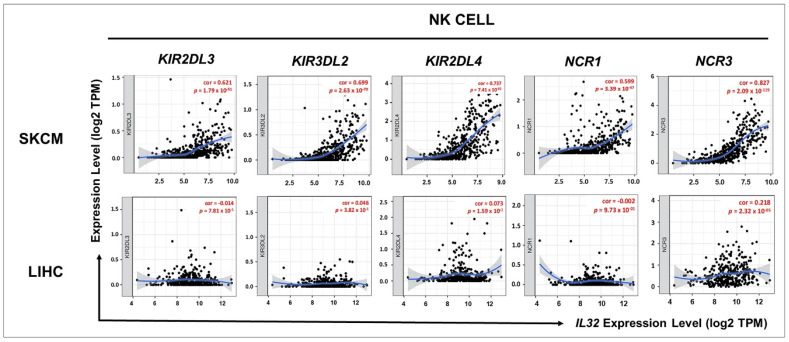
Correlation of *IL32* expression with NK markers. The correlation between *IL32* expression and various gene markers of NK was examined using the TIMER v.1.0 web tool. The *KIR2DL3*, *KIR3DL2, KIR2DL4, NCR1*, and *NCR3* genes were used as gene markers for NK cells. *IL32* expression is positively related to the expression of gene markers for NK cells in SKCM. *IL32* expression is not significantly correlated with the expression of most gene markers in LIHC. Supplementary Table S10 provides the *p*-values and correlation constants.

To further investigate the correlation between *IL32* expression and various subsets of immune cell infiltrations in SKCM, we analyzed the correlations between *IL32* and immune cell markers, including subsets of each immune cell in SKCM (Supplementary Table S9 and Figure 5). Supplementary Table S9 shows the immune cell markers including subsets of T cells (general T cells, CD8^+^ T cells, CD4^+^ T cells, regulatory T cells, and T cell exhaustion), B cells, monocytes, NK cells, TAM, M1 and M2 macrophages, and neutrophils in SKCM. These data reveal that *IL32* expression is correlated significantly with most of the immune marker genes of NK cells in SKCM and that *IL32* expression was significantly correlated with NK cells in SKCM but not in LIHC. Therefore, to examine the association between *IL32* expression and the infiltration of NK cell subsets, the correlation between *IL32* expression and gene marker expression in each immune cell was examined in SKCM. As shown in Figure 5, *IL32* expression was positively correlated with *KIR2DL3* (cor = 0.621, *p* = 1.79 × 10^−51^), *KIR3DL2* (cor = 0.699, *p* = 2.63 × 10^−70^), *KIR2DL4* (cor = 0.737, *p* = 7.41 × 10^−82^), *NCR1* (cor = 0.599, *p* = 3.39 × 10^−47^), and *NCR3* (cor = 0.827, *p* = 2.09 × 10^−119^) gene expressions in SKCM, whereas *IL32* expression was not significantly correlated with the expression of gene markers in LIHC (*KIR2DL3*, cor = −0.014, *p* = 7.81 × 10^−1^; *KIR3DL2*, cor = 0.046, *p* = 3.82 × 10^−1^; *KIR2DL4*, cor = 0.073, *p* = 1.59 × 10^−1^; *NCR1*, cor = −0.002, *p* = 9.73 × 10^−1^; and *NCR3*, cor = 0.218, *p* = 2.32 × 10^−5^). Moreover, Figure 5 and Table 1 show that the expression of *IL32* and the expression of NK cell markers are not correlated in LIHC but are highly correlated in SKCM, which is also shown in data from the TIMER database. Taken together, these data suggest that *IL32* expression is markedly correlated with infiltrated NK cells in SKCM.

### 3.6. Correlation between IL32 Expression and Activation of NK Cells

**Figure 6:**
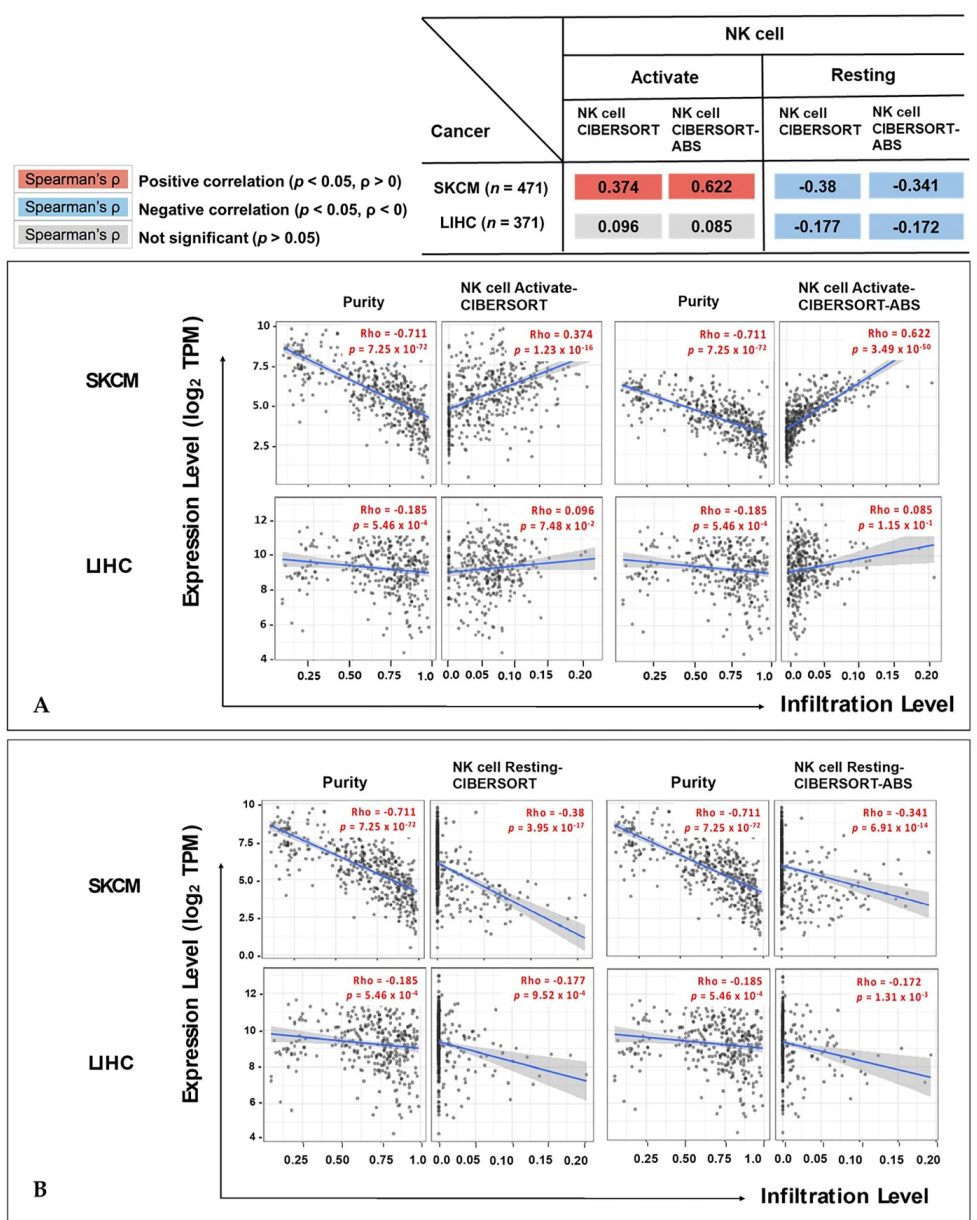
Correlation between *IL32* expression and the infiltration levels of NK cells in SKCM and LIHC. The relationship between expression levels of *IL32* and (**A**) activated NK cells, and (**B**) resting NK cell was investigated by the online tool TIMER v.2.0. (*p* < 0.05).

To confirm the difference between activating and resting NK cell infiltration by *IL32* expression, we analyzed the expression of activated NK cells from CIBERSORT using TIMER v.2.0. As shown in Figure 6A, a high *IL32* expression was positively correlated with the infiltration of activated NK cells (rho = 0.374, *p* = 1.23 × 10^−16^) in SKCM, but no correlation was found between *IL32* expression in LIHC (activated NK cells; rho = 0.096, *p* = 7.48 × 10^−2^). Interestingly, the infiltration of resting NK cells showed a significant negative correlation with *IL32* expression in SKCM (rho = −0.38, *p* = 3.95 × 10^−17^) and LIHC (rho = −0.177, *p* = 9.52 × 10^−4^) (Figure 6B). We further analyzed NK cell infiltration according to SKCM status to prove the correlation between *IL32* expression and survival. These results suggest that *IL32* expression in SKCM tissue induces the infiltration of specially activated NK cells. Overall, in this study, *IL32* expression controlled activated NK cell infiltration in SKCM and improved the prognosis of melanoma patients.

### 3.7. Correlation between IL32 Expression and Cytolytic Cell of NK Cells

**Figure 7:**
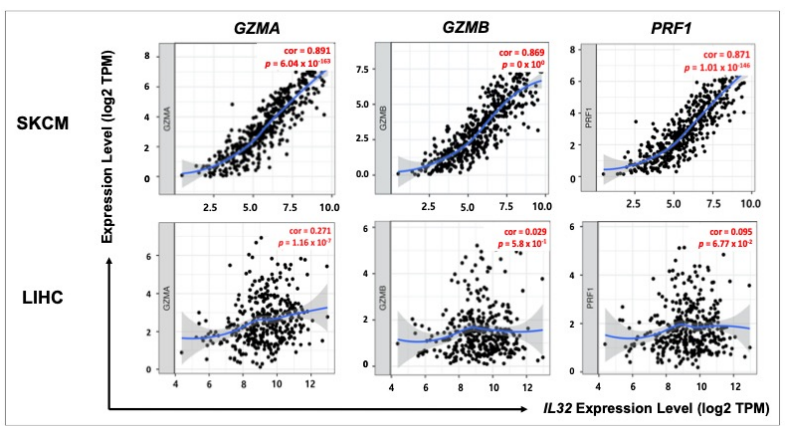
Correlation between *IL32* expression and cytolytic effector cells in SKCM and LIHC. Scatter plots were generated using TIMER v.1.0. *IL32* expression and gene markers of the cytolytic effector were positively correlated in SKCM. *IL32* expression was not significantly correlated with either gene in LIHC. The correlation constants and *p*-values are listed in Supplementary Table S11. (*p* < 0.05).

NK cells are well-known representative cytolytic effector cells that release cytotoxic molecules, such as granzyme and perforin [52]. Therefore, we analyzed the correlation between *IL32* expression and gene markers of the cytolytic molecules granzyme A (*GZMA*)*,* granzyme B *(GZMB*), and perforin (*PRF1*) using TIMER v.1.0. As shown in Figure 7, *IL32* expression was significantly positively correlated with *GZMA* (cor = 0.891, *p* = 6.04 × 10^−163^), *GZMB* (cor = 0.869, *p* = 0 × 10^0^), and *RPF1* (cor = 0.871, *p* = 1.01 × 10^−146^) gene expressions in SKCM (*n* = 103), but no correlation was found between *IL32* expression and gene markers of cytolytic molecules in LIHC (*n* = 371) (*GZMA*, cor = 0.271, *p* = 1.16 × 10^−7^; *GZMB*, cor = 0.029, *p* = 5.8 × 10^−1^; and *RPF1*, cor = 0.095, *p* = 6.77 × 10^−2^). Collectively, these results suggest that infiltrating NK cells improve SKCM patient survival by impeding tumor progression via the release of granzyme and perforin.

### 3.8. Coexpressed Genes and IL32 in Melanoma

**Figure 8:**
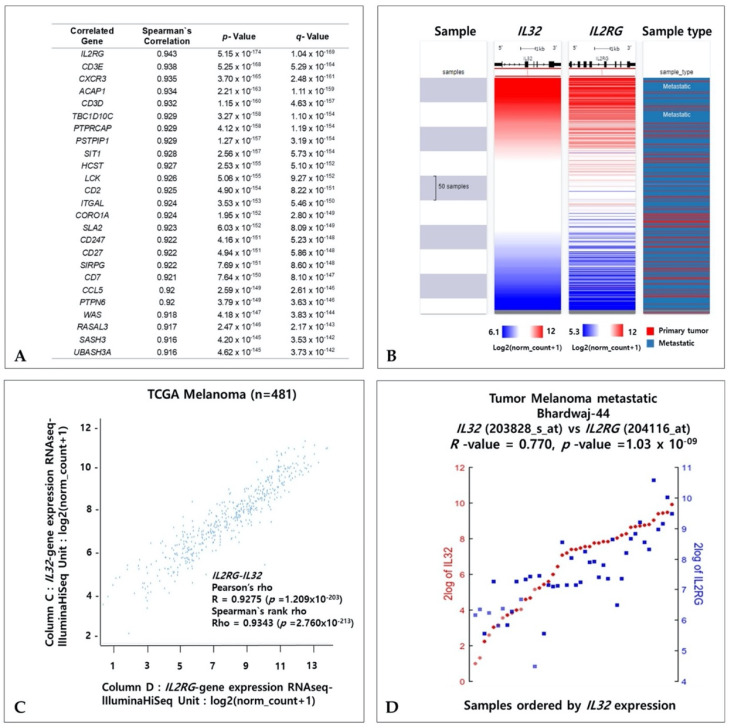
Coexpression genes of the *IL32* gene in SKCM. (**A**) Twenty-five genes most positively coexpressed with *IL32* in SKCM using the TCGA dataset of cBioPortal. (**B**) *IL32* and *IL2RG* mRNA expressions in a heatmap using the UCSC Xena Browser. (**C**) Dot plot of *IL32* and *IL2RG* mRNA expressions in the TCGA-SKCM dataset. (**D**) Correlation between *IL32* and *IL2RG* expressions in the Tumor Melanoma Metastatic Bhardwaj-44 dataset using the R2 web server 3. (*p* < 0.05).

To investigate the coexpressed genes with *IL32* in SKCM, we identified genes that exhibit correlated expressions with *IL32* in SKCM using the TCGA dataset of cBioPortal. Figure 8A shows that 25 genes are the most positively coexpressed with *IL32* in SKCM. In the TCGA-SKCM dataset, the gene expression of the *interleukin 2 receptor group* (*IL2RG*) showed the strongest positive correlation with *IL32*. The coexpressed patterns of *IL32* and *IL2RG* were analyzed via a heatmap and a dot plot using the UCSC Xena web tool (Figure 8B,C). The coexpression patterns of *IL32* and *IL2RG* in primary and metastatic melanomas were visually expressed through a heatmap (Figure 8B). In Figure 8C, the strong positive correlation between *IL32* and *IL2RG* expression was confirmed using Pearson’s (R = 0.9275, *p* = 1.209 × 10^−203^) and Spearman’s (R = 0.9343, *p* = 2.76 × 10^−213^) correlation analyses in primary and metastatic SKCM. Additionally, the correlations with *IL32* and *IL2RG* were confirmed using the R2 platform (Tumor melanoma metastatic Bhardwaj-44, R = 0.770, *p* = 1.03 × 10^−9^) (Figure 8D). Taken together, these data suggest that *IL32* and coaltered *IL2RG* with biological processes related to IL-32 may be involved in melanoma progression.

**Figure 9:**
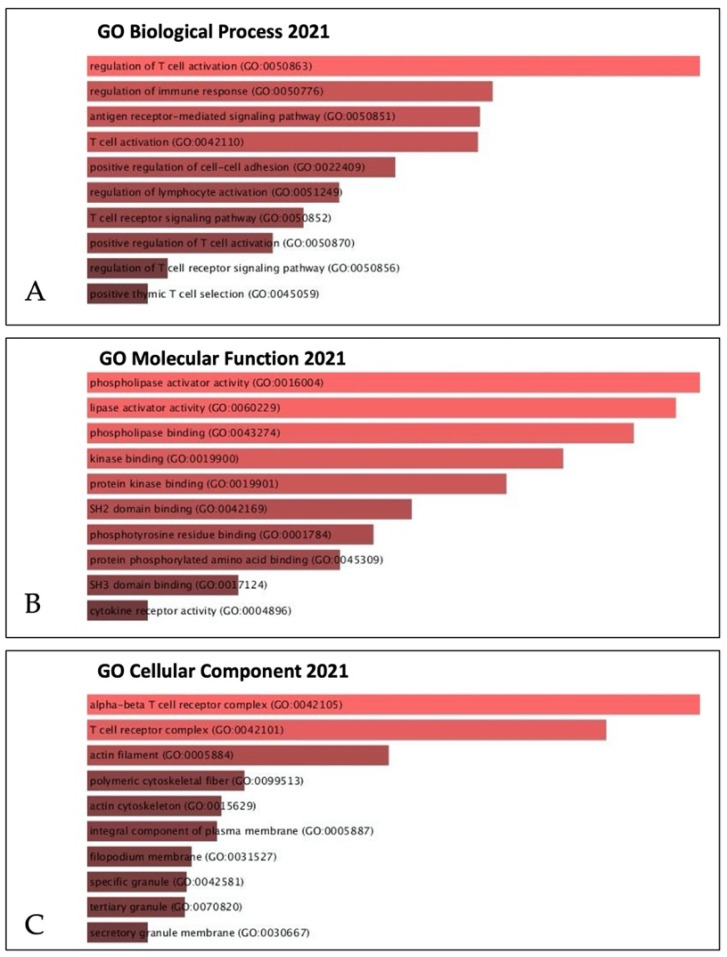
Correlation between *IL32* gene and coexpression genes in the SKCM signaling pathway. *IL32* gene and its top 25 coexpressed genes were analyzed by the Enricher web tool (https://amp.pharm.mssm.edu/Enrichr/, accessed on 25 April 2021). (**A**) GO biological process (2021), (**B**) GO molecular function (2021), and (**C**) GO cellular component (2021).

In addition, to identify biological processes and functions, we further analyzed the correlation between *IL32* and coaltered genes in SKCM using the gene ontology (GO) analysis (Figure 9). In the GO biological process analysis, *IL32* and *IL32* coaltered genes were mainly associated with regulated immne response (Figure 9A). In the GO molecular function analysis, *IL32* coaltered genes were most significantly enriched in phospholipase cativator activity, and lipase activator and phospholipase binding (Figure 9B). Figure 9C showed the most abundant results in the GO cell components associated with the alpha–beta T cell receptor complex (Figure 9C). Overall, a gene enrichment analysis of *IL32* and *IL32* coaltered genes suggests that *IL32* may be associated with the regulation of lymphocyte activation.

## 4. Discussion

IL-32 is a proinflammatory cytokine involved in various isoforms and is known to have both anticancer and procancer properties [53]. To date, nine isoforms have been identified, and the four most studied isoforms, IL-32α, IL-32β, IL-32γ, and IL-32δ, were first described in NK cells [38,54]. All of these isoforms in IL-32 have different sizes and secondary structures, which can lead to differences in protein function and efficacy due to changes in the tertiary protein structure [55]. Indeed, various isoforms of IL-32 show differences in efficacy to elicit a specific effect and to induce different reactions in malignant tumors [56,57]. Exogenous treatment with IL-32α inhibited proliferation and increased apoptosis in HTB-72 human melanoma cell lines in relation to the upregulation of p21, p53, and TRAIL receptor 1 (TRAILR1) [58]. However, the high expression of IL-32β was increased in cancer tissues and serum in patients with hepatocellular carcinoma, and the inhibition of IL-32α expression using siRNA resulted in decreased expression of the antiapoptotic protein bcl-2, inhibiting cell growth and apoptosis [59]. In addition, Oh et al. showed that IL-32β and IL-32γ inhibit melanoma and colon cancer tumor growth via inhibition of the activated NF-κB and STAT3 in a transgenic mouse model, while the expression of these isoforms is associated with increased infiltration and migration of breast cancer cells [43,60,61]. Although many studies have revealed the mechanism of the role of IL-32 isoforms in various types of cancers, the role of IL-32 is controversial and still unclear.

Recently, *IL32* expression and cancer-related immune cells have been reported to be highly correlated in various types of cancers [62,63]. *IL32* is produced by representative antitumor immune cells such as T cells, NK cells, monocytes, and macrophages [34,38]. In colorectal and prostate cancer cells, IL-32β has been shown to enhance NK cytotoxicity against cancer cells in vitro via the activation of caspase-3 [64]. In particular, IL-32β expression in metastatic mice increases the level of IL-10, an immunosuppressive cytokine, and induces infiltration of cytotoxic T cells and NK cells in tumors, resulting in the suppression of tumor growth [61]. These results suggest that IL-32 may stimulate antitumor immune responses in tumor microenvironments by inducing cytolytic activity and the infiltration of NK cells and T cells. Although various biological activities of IL-32 in tumor progression have been reported, a comprehensive analysis is required in clinical studies along with in vitro and in vivo studies because IL-32 has dual effects in tumor biology, such as procancer effects and anticancer effects. Therefore, a systematic analysis of the correlation between *IL32* expression and patient survival is essential to comprehensively understand the role of IL-32 in melanoma patients.

Here, we found that *IL32* mRNA expression was higher in cutaneous melanoma (SKCM; skin cutaneous melanoma) tissue than in normal tissue and that a higher expression of *IL32* was significantly correlated with patient survival (Figure 1 and Figure 2), suggesting that a higher *IL32* expression leads to better clinical outcomes in SKCM patients. Our analysis also showed that *IL32* expression levels were positively correlated with the levels of infiltration of various immune cells, especially NK cells (Figure 4). The fact that high infiltration levels of immune cells result in better prognosis in various types of cancers is well known [65]. As shown in Figure 5, a strong negative correlation was found between *IL32* expression and tumor purity, indicating that *IL32* mRNA expression in SKCM tissues is likely to have been derived from infiltrated immune cells. Therefore, the reason why the patient’s survival rate increases with the increase in *IL32* mRNA expression in SKCM tissues can be presumed to be due to the infiltration of activated NK cells expressing *IL32*. In fact, *IL32* was mainly detected in activated NK cells and dendritic cells (DCs), which induce tumor cell apoptosis by producing cytolytic molecules such as perforin and granzyme from activated NK cells in tumor environments [66,67]. Nevertheless, the fact that the activities and functions of effector cells in the TME are suppressed is known [68,69]. Tumor cells secrete immunosuppressive cytokines such as IL-10, or immune cells, such as regulated T cells and M2 macrophages in the TME, inhibit the activity of antitumor effector cells due to their immune inhibition function [52,70]. Therefore, in immunotherapy, maintaining the activity of immune cells and increasing the infiltration of effector cells in tumors are strongly related to better outcomes in patients. In this regard, this study attempted to determine whether the increase in patient survival rate following *IL32* mRNA expression was due to the infiltration of activated effector cells. Figure 5 and Supplementary Table S10 show a strong positive correlation between *IL32* expression and the expression of specific markers for NK cells. Interestingly, the expression of *IL32* and resting NK cell infiltration were negatively correlated while activated NK cell infiltration was positively correlated (Figure 6). These results indicate that activated NK cells were infiltrated within the tumor of SKCM and that tumor suppression was induced through the activation of NK cell cytotoxicity. In fact, this study identified a positive correlation between *IL32* expression and cytolytic molecule genes such as *GZMA, GZMB,* and *PRF1* (Figure 7). This suggests that *IL32* could increase patient survival by enhancing the infiltration and cytolytic activities of NK cells in effector cells such as granzyme and perforin. Therefore, our study found that *IL32* expression levels are correlated with NK cell infiltration in SKCM.

In addition, we investigated the co-expressed genes with biological processes related to *IL32* in SKCM. Of the 25 genes that were positively correlated, IL-2 receptor γ (*IL2RG*) showed the strongest positive correlation with *IL32* expression as shown in Figure 8. IL2RG is well known as a common subunit for the signaling of various interleukins, including IL-2, -4, -7, -9, and -15. It is essential for affinity binding and signaling of cytokines and plays an important role in the development and survival of immune cell subgroups, such as NK cells and T cells [71]. In addition, Figure 9 shows that *IL32* is involved in the regulation of lymphocyte activation by GO analysis. Therefore, these data suggest that *IL32* and *IL32*-coaltered genes are involved in antitumor immune regulation by immune cells, including NK cells, and that *IL32* expression may be a novel biomarker for predicting immune cell activation in SKCM.

## 5. Conclusions

In conclusion, this study shows that the increased *IL32* mRNA expression is significantly related to the infiltration of NK cells in cutaneous melanoma tissues, resulting in a good prognosis in cutaneous melanoma patients. The main finding of this study is that the correlation between *IL32* mRNA expression and activated NK cell infiltration is significant but that the correlation between *IL32* mRNA expression and a resting NK cell is not. In addition, the correlation between *IL32* and various genes of cytolytic molecules, such as *GZMA, GZMB,* and *PRF1*, is positive, suggesting that *IL32* mRNA expression may increase patient survival through the infiltration and activation of anticancer effector cells in cutaneous melanoma. This systematic analysis provides evidence suggesting the potential role of *IL32* as an effective biomarker for patient survival in the tumor microenvironment.

## Figures and Tables

**Table 1:** Correlation between *IL32* and NK cell markers in GEPIA2.

	Gene Markers	SKCM		LIHC	
		R	*p*	R	*p*
NK cell	*KIR2DL1*	0.33	***	−0.0046	0.93
	*KIR2DL3*	0.35	***	−0.055	0.29
	*KIR2DL4*	0.6	0	0.056	0.28
	*KIR3DL1*	0.37	0	0.018	0.72
	*KIR3DL2*	0.58	0	−0.024	0.64
	*KIR3DL3*	0.14	*	0.029	0.57
	*KIR2DS4*	0.21	***	−0.052	0.32
	*KLRK1 (NKG2D)*	0.69	0	0.065	0.21
	*NCR1 (NKp46)*	0.33	***	−0.039	0.46
	*NCR2 (NKp44)*	0.11	0.019	−0.025	0.63
	*NCR3 (NKp30)*	0.47	0	0.09	0.085

* *p* < 0.01 and *** *p* < 0.0001.

## Data Availability

Publicly available datasets were analyzed in this study. This data can be found in the link mentioned in the section of Materials and Methods.

## Supplementary Materials

The following are available online at https://www.mdpi.com/article/10.3390/jcm10204691/s1, Supplementary Table S1. The Cox regression results of IL32 expression in SKCM and LIHC; Table S2. Tumor abbreviations; Figure S3. Cox regression results for IL32 with TCGA data in various types of cancers; Table S4. Correlation between IL32 expression and DFS of various types of cancers; Table S5. Fifteen mutations in the IL32 gene; Figure S6. Correlation IL32 expression and BRAF mutation status in SKCM; Figure S7. Correlation of IL32 expression with immune cell infiltration level in SKCM and LIHC; Figure S8. Correlations between IL32 expression and NK cells of various types of cancers; Table S9. Correlation analysis between IL32 and gene markers of immune cells; Table S10. Correlation constants and *p*-values in Figure 5; Table S11. Correlation constants and *p*-values in Figure 7.
