# Supplementary material for: Prognostic Value of Interleukin-32 Expression and Its Correlation with the Infiltration of Natural Killer Cells in Cutaneous Melanoma
Source: J Clin Med. 2021 Oct 13;10(20):4691. doi: 10.3390/jcm10204691 (PMC8538574; doi:10.3390/jcm10204691)

Supplementary Table S1. The cox regression results of *IL32* expression in SKCM and LIHC.

KM plot data using TIMER version 1.0 (p < 0.05)

	SKCM						LIHC					
	coef	HR	95% CI _low	95% CI _upper	*P*-value	significant	coef	HR	95% CI _low	95% CI _upper	*P*-value	significant
**Purity**	−0.218	0.804	0.339	1.907	0.621		0.487	1.627	.660	4.012	0.290	
**IL32**	−0.252	0.777	0.691	0.874	0.000	***	−0.103	0.903	0.784	1.039	0.155	
**Age**	0.018	1.018	1.008	1.029	0.000	***	0.011	1.011	0.995	1.027	0.174	
**Stage2**	0.131	1.140	0.736	1.765	0.558		0.312	1.367	0.811	2.305	0.241	
**Stage3**	0.528	1.696	1.134	2.538	0.010	*	0.944	2.57	1.623	4.067	0	***
**Stage4**	1.352	3.866	1.935	7.725	0	***	1.582	4.862	1.441	16.402	0.011	*
**Gender**	−0.065	0.937	0.686	1.279	0.682		−0.101	0.904	0.574	1.424	0.664	
**Race**	−1.386	0.250	0.114	0.550	0.001	**	−0.031	0.969	0.608	1.546	0.896	

*p < 0.01; **p < 0.001; ***p < 0.0001.

KM plot data using TIMER version 2.0 (p < 0.05, Z< 0)

	SKCM								LIHC							
	coef	HR	Se (coef)	95% CI _low	95% CI _upper	Z-value	*P*-value	significant	coef	HR	Se (coef)	95% CI _low	95% CI _upper	Z-value	*P*-value	significant
**Purity**	−0.203	0.816	0.442	0.343	1.94	−0.46	0.645		0.52	1.683	0.458	0.686	4.129	1.136	0.256	
**IL32**	−0.251	0.778	0.06	0.691	0.875	−4.175	0	***	−0.097	0.908	0.071	0.789	1.044	−1.352	0.176	
**Age**	0.018	1.018	0.005	1.008	1.029	3.393	0.001	**	0.012	1.012	0.008	0.997	1.028	1.523	0.128	
**Stage2**	0.111	1.117	0.224	0.72	1.734	0.495	0.621		0.319	1.376	0.262	0.823	2.3	1.219	0.223	
**Stage3**	0.522	1.686	0.206	1.127	2.523	2.54	0.011	*	0.944	2.57	0.234	1.623	4.067	4.028	0	***
**Stage4**	1.352	3.866	0.353	1.935	7.714	3.824	0	***	1.55	4.71	0.619	1.399	15.862	2.502	0.012	*
**Gender**	−0.075	0.928	0.159	0.679	1.268	−0.469	0.639		−0.091	0.913	0.029	0.583	1.43	−0.397	0.691	
**Race**	−1.401	0.246	0.403	0.112	0.542	−3.48	0.001	**	−0.001	0.999	0.236	0.629	1.587	−0.005	0.996	

*$p < 0.01$; **$p < 0.001$; ***$p < 0.0001$.

Supplementary Table S2. Statistical tests used in Figures and Supplementary data.

Figure	Database	Object	Method	Sample	Statistical test	*p*-value ($p < 0.05$)
**Figure 1**	GEPIA2	mRNA Expression level	TCGA & GTEx projects	Tumor ($n = 461$)<br>Normal ($n = 558$)	ANOVA<br>Empirical Bayes method	$p = 3.5×10^{-63}$
		Box plot			One-way ANOVA	
**Figure 2**	GEPIA2	Survival plot (Overall)	TCGA & GTEx projects	High ($n = 229$)<br>Low ($n = 229$)	Log rank test (Mantel-Cox test)	$p = 3.1×10^{-4}$
	TIMER 1.0		TCGA	$n = 425$	Proportional hazards model	$p = 0×10^{+00}$
	TIMER 2.0		TCGA cohorts	$n = 471$	Proportional hazards model	$p = 4.6×10^{-2}$
**Figure 3**	UALCAN	Promoter methylation	TCGA	Normal ($n = 2$)<br>Primary ($n = 104$)<br>Metastasis ($n = 368$)	Student`s t-test	Nor vs Pri ($p = 4.23×10^{-1}$)<br>Nor vs Met ($p = 5.95×10^{-5}$)<br>Pri vs Met ($p = 9.15×10^{-1}$)
**Figure 4**	TISIDB	Expression of immune cells	gene set variation analysis (GSVA)	$n = 472$	Empirical bayes estimate	$p = 2.2×10^{-6}$
**Figure 5**	TIMER Version 1.0	Correlation	TCGA	$n = 103$	Bayesian statistics<br>Gibbs sampling<br>Expectation maximization algorithm	KIR2DL3 ($p = 1.79×10^{-11}$)<br>KIR3DL2 ($p = 2.63×10^{-70}$)<br>KIR2DL4 ($p = 7.41×10^{-82}$)<br>NCR1 ($p = 3.39×10^{-47}$)<br>KNCR3 ($p = 2.09×10^{-119}$)
**Figure 6**	TIMER Version 2.0	Correlation	CIBERSORT	$n = 471$	Bayesian statistics<br>Gibbs sampling<br>Expectation maximization algorithm	Purity ($p = 7.25×10^{-72}$)<br>Activated NK cells ($p = 1.23×10^{-16}$)<br>Rested NK cells ($p = 3.95×10^{-17}$)
			CIBERSORT-ABS		Bayesian statistics<br>Gibbs sampling<br>Expectation maximization algorithm	Purity ($p = 7.25×10^{-72}$)<br>Activated NK cells ($p = 3.49×10^{-50}$)<br>Rested NK cells ($p = 6.91×10^{-14}$)

**Figure 7**	TIMER<br>Version 1.0	Correlation	TCGA	$n = 103$	Bayesian statistics<br>Gibbs sampling<br>Expectation maximization algorithm	GZMA ($p = 6.04\times10^{-163}$)<br>GZMB ($p = 0\times10^{+00}$)<br>PRF1 ($p = 1.01\times10^{-146}$)
**Figure 8**	cBioportal	Co-expressed gene list	TCGA	-	Clustering statistics	*IL2RG* ($p = 5.15\times10^{-174}$)
	UCSC Xena	Correlation	TCGA	$n = 481$		*IL32-IL2RG* ($p = 7.76\times10^{-213}$)
	R2	Correlation	-	$n = 44$	Brute force t-test<br>One way ANOVA	*IL32-IL2RG* ($p = 1.03\times10^{-9}$)
**Figure 9**	Enricher	GO biological process	-	-	Standard statistical method<br>Fisher's exact test<br>Hypergeometric test	Biological process ($p = 1.49\times10^{-5}$)<br>Molecular Function ($p = 8.19\times10^{-5}$)<br>Cellular component ($p = 3.44\times10^{-8}$)
**Supplementary S4**	OncoLnc	Cox regression	-	-	Cox proportional Hazards model	$p = 7.90\times10^{-4}$
**Supplementary S5**	GEPIA2	Survival plot (Disease Free Survival)	TCGA &<br>GTEx projects	High ($n = 229$)<br>Low ($n = 229$)	Log rank test<br>(Mantel-Cox test)	$p = 2.0\times10^{-1}$
**Supplementary S7**	TIMER<br>Version 2.0	Correlation (BRAF)	TCGA	$n = 465$	Bayesian statistics<br>Gibbs sampling<br>Expectation maximization algorithm	$p = 6.4\times10^{-2}$
**Supplementary S8**	TIMER<br>Version 1.0	Correlation	TCGA	$n = 103$	Bayesian statistics<br>Gibbs sampling<br>Expectation maximization algorithm	Purity ($p = 7.25\times10^{-72}$)<br>B cell ($p = 3.66\times10^{-11}$)<br>CD8+ T cell ($p = 1.05\times10^{-31}$)<br>CD4+ T cell ($p = 2.05\times10^{-22}$)<br>Macrophage ($p = 1.00\times10^{-10}$)<br>Neutrophill ($p = 1.32\times10^{-32}$)<br>Dendritic cell ($p = 6.01\times10^{-58}$)
**Supplementary S13**	GEPIA2	Correlation	TCGA &<br>GTEx projects	Tumor ($n = 461$)	Spearman correlation	CXCL9 ($p = 1.7\times10^{-112}$)<br>CXCL10 ($p = 3.0\times10^{-77}$)<br>CXCL11($p = 3.0\times10^{-68}$)

Supplementary Table S3. Tumor Abbreviations

Abbreviation	Type of Cancer
**ACC**	Adrenocortical carcinoma
**BLCA**	Bladder Urothelial Carcinoma
**BRCA**	Breast invasive carcinoma
**CESC**	Cervical squamous cell carcinoma and endocervical adenocarcinoma
**CHOL**	Cholangio carcinoma
**COAD**	Colon adenocarcinoma
**DLBC**	Lymphoid Neoplasm Diffuse Large B-cell Lymphoma
**ESCA**	Esophageal carcinoma
**GBM**	Glioblastoma multiforme
**HNSC**	Head and Neck squamous cell carcinoma
**KICH**	Kidney Chromophobe
**KIRC**	Kidney renal clear cell carcinoma
**KIRP**	Kidney renal papillary cell carcinoma
**LAML**	Acute Myeloid Leukemia
**LGG**	Brain Lower Grade Glioma
**LIHC**	Liver hepatocellular carcinoma
**LUAD**	Lung squamous cell carcinoma
**MESO**	Mesothelioma
**OV**	Ovarian serous cystadenocarcinoma
**PAAD**	Pancreatic adenocarcinoma
**PCPG**	Pheochromocytoma and Paraganglioma
**PRAD**	Prostate adenocarcinoma
**READ**	Rectum adenocarcinoma
**SARC**	Sarcoma
**SKCM**	Skin Cutaneous Melanoma
**STAD**	Stomach adenocarcinoma
**TGCT**	Testicular Germ Cell Tumors
**THCA**	Thyroid carcinoma

Supplementary Table S4. Cox regression results for *IL32* with TCGA data in various types of cancers by OncoLnc. The data with *p*-value < 0.01 are marked with red.

Cancer	Cox Coefficient	*p*-Value	FDR Corrected	Rank	Median Expression	Mean Expression
SKCM	−0.224	7.90×10^{-4}	1.20×10^{-2}	1059	630.9	1326.15
SARC	−0.362	1.50×10^{-3}	4.07×10^{-2}	586	1220.88	2354.81
PAAD	0.26	1.90×10^{-2}	1.06×10^{-1}	3030	4441.18	4962.08
LIHC	−0.168	8.30×10^{-2}	2.92×10^{-1}	4498	11949.06	21242.03
UCEC	−0.152	1.50×10^{-1}	9.68×10^{-1}	2492	2084.05	3466.36
BLCA	−0.104	1.70×10^{-1}	4.19×10^{-1}	6528	1100.77	2227.77
BRCA	−0.101	2.30×10^{-1}	6.10×10^{-1}	6186	904.08	1452.49
OV	−0.092	2.40×10^{-1}	7.95×10^{-1}	5005	810.49	1177.3
LUAD	0.085	2.60×10^{-1}	5.04×10^{-1}	8573	2344.86	2989.48
KIRP	−0.161	2.90×10^{-1}	4.76×10^{-1}	9943	10570.44	12843.81
HNSC	−0.073	3.10×10^{-1}	6.43×10^{-1}	7923	2205.22	3464.29
GBM	0.092	3.30×10^{-1}	8.85×10^{-1}	6182	346.67	436.5
KIRC	−0.078	3.40×10^{-1}	4.71×10^{-1}	12002	7784.6	9252.17
CESC	−0.108	3.80×10^{-1}	7.11×10^{-1}	8667	3097.67	4733.14
LUSC	0.062	3.80×10^{-1}	8.17×10^{-1}	7818	1318.88	2071.46
LGG	0.062	5.10×10^{-1}	6.07×10^{-1}	14107	155.38	260.48
READ	−0.114	6.10×10^{-1}	9.77×10^{-1}	10215	5257.51	5898.36
LAML	−0.058	6.40×10^{-1}	8.71×10^{-1}	11143	176.05	295.76
STAD	−0.038	6.40×10^{-1}	8.82×10^{-1}	12202	2537.94	3540.33
COAD	−0.043	6.90×10^{-1}	9.02×10^{-1}	12490	5428.92	6129.97
ESCA	−0.04	7.70×10^{-1}	9.85×10^{-1}	13041	1322.23	1910.35

Supplementary Figure S5. Correlation between *IL32* expression and disease free survival (DFS) of various types of cancers by GEPIA2. (log-rank $p < 0.05$)

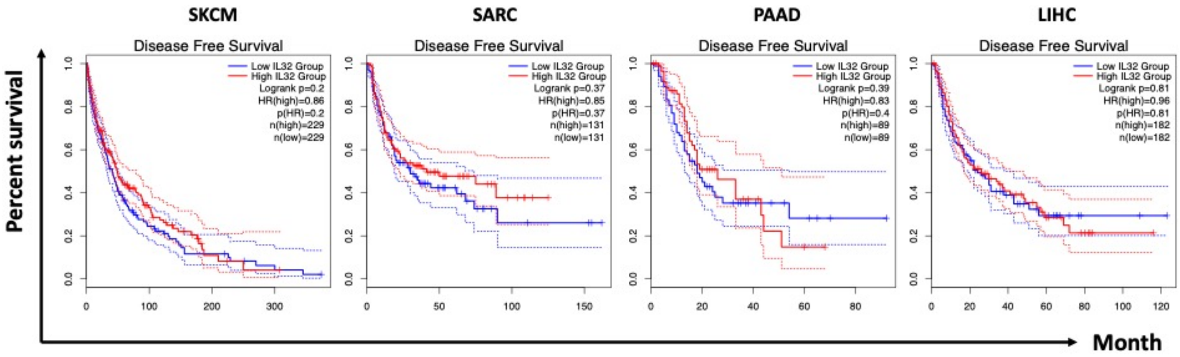

Supplementary Table S6. Fifteen mutations in the *IL32* gene by cBioportal.

Sample ID	Protein Change	Mutation Type	Copy #	Allele Freq (T)	# Mut in Sample
TCGA-W3-AA1W–06	X10_splice	Splice	Diploid	0.19	586
TCGA-W3-AA1V–06	R70K	Missense	Diploid	0.57	4559
TCGA-W3-AA1V–06	P224L	Missense	Diploid	0.09	4559
TCGA-FW-A3R5–06	S133F	Missense	ShallowDel	0.17	15825
TCGA-FW-A3R5–06	D140N	Missense	ShallowDel	0.29	15825
TCGA-FW-A3R5–06	M155I	Missense	ShallowDel	0.28	15825
TCGA-FS-A1ZZ–06	S34L	Missense	Diploid	0.45	1530
TCGA-ER-A193–06	K234I	Missense	ShallowDel	0.31	1700
TCGA-EE-A2MS–06	K121*	Nonsense	Diploid	0.25	2462
TCGA-EE-A2GO–06	T38I	Missense	ShallowDel	0.28	1909
TCGA-EE-A17X–06	F150S	Missense	Diploid	0.26	680
TCGA-D9-A6EC–06	F3V	Missense	ShallowDel	0.3	3676
TCGA-D3-A51F–06	X10_splice	Splice	Diploid	0.06	103
TCGA-D3-A2JF–06	P53L	Missense	Diploid	0.43	1213
TCGA-D3-A2J8–06	X10_splice	Splice	Diploid	0.1	953

Supplementary Figure S7. Correlation *IL32* expression and BRAF mutation status in SKCM by TIMER v.2.0. ($p < 0.05$)

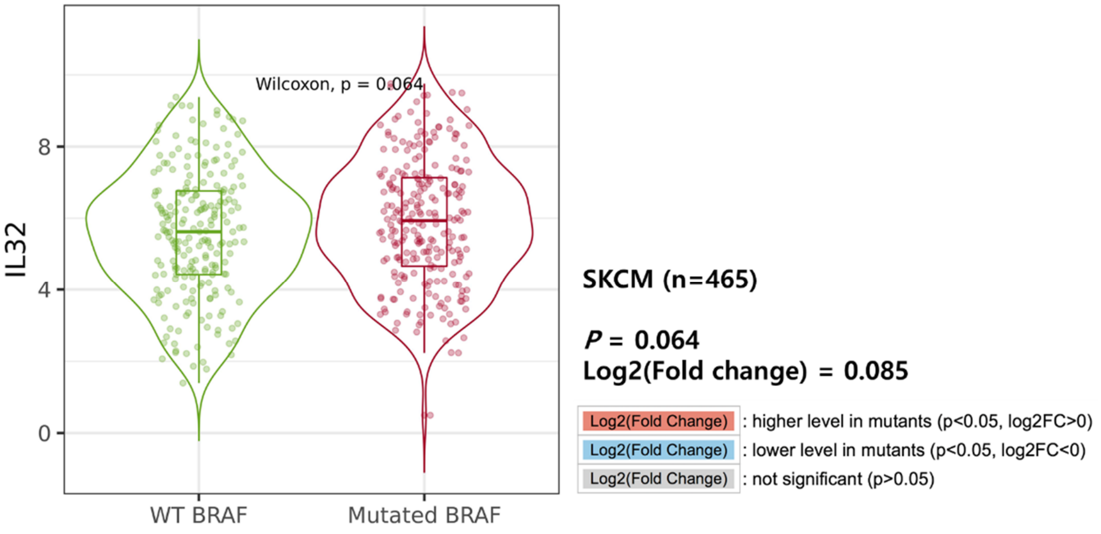

Supplementary Figure S8. Correlation of *IL32* expression with immune cell infiltration level in SKCM and LIHC. (TIMER v.1.0) ($p < 0.05$)

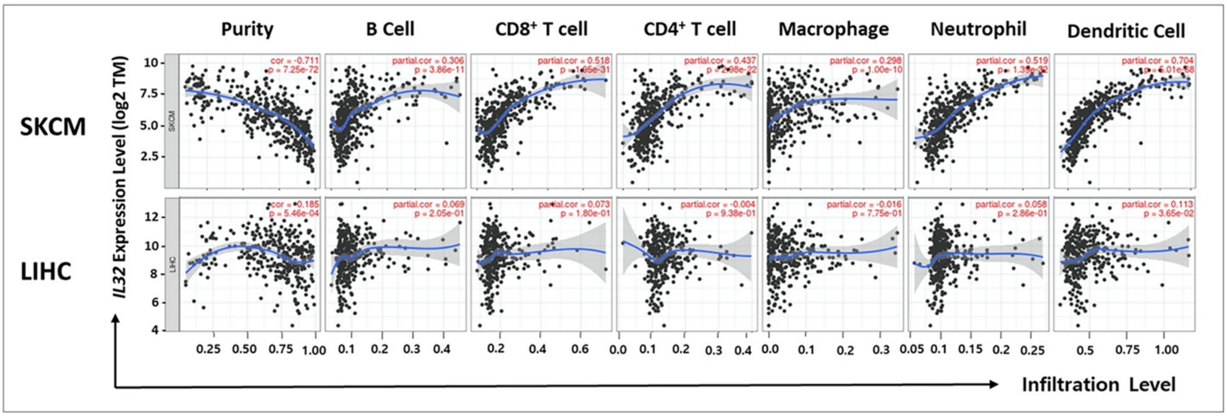

Supplementary Figure S9. Correlations between *IL32* expression and NK cells of various types of cancers by TISIDB. ($p < 0.05$)

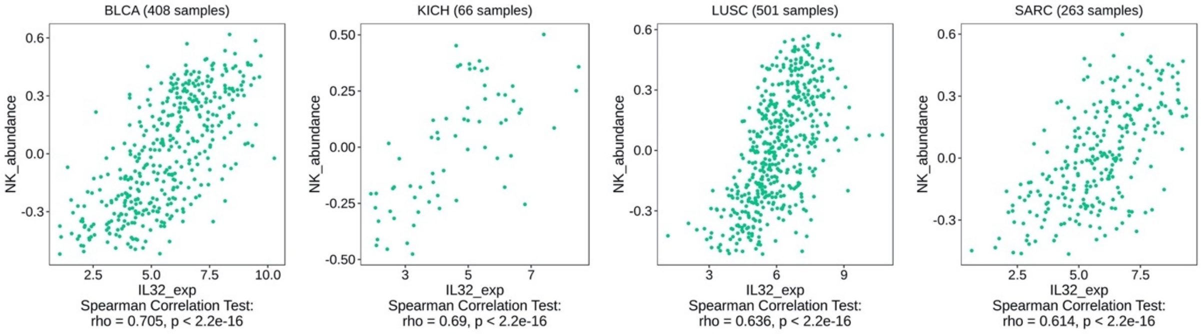

Supplementary Table S10. Correlation analysis between *IL32* and gene markers of immune cells using TIMER (v.1.0).

Description	Gene markers	SKCM				LIHC			
		None		Purity		None		Purity	
		Cor	*P*	Cor	*P*	Cor	*P*	Cor	*P*
Natural killer cell	*KIR2DL1*	0.459	***	0.32	***	−0.048	0.35	−0.066	0.23
	KIR2DL3	0.621	***	0.46	***	−0.014	0.78	−0.051	0.35
	KIR2DL4	0.737	***	0.63	***	0.073	0.16	0.038	0.49
	KIR3DL1	0.618	***	0.49	***	0.006	0.91	−0.028	0.60
	KIR3DL2	0.699	***	0.55	***	0.046	0.38	−0.022	0.68
	KIR3DL3	0.243	***	0.19	***	0.000	1.00	−0.022	0.68
	KIR2DS4	0.513	***	0.40	***	−0.047	0.37	−0.059	0.27
	NCR1	0.599	***	0.487	***	−0.002	0.97	−0.065	0.22
	NCR3	0.827	***	0.712	***	0.218	***	0.137	***

CD8+ T cell	*CD8A*	0.869	0	0.80	***	0.170	***	0.081	0.135
	CD8B	0.882	***	0.81	***	0.138	***	0.044	0.412
Treg	*FOXP3*	0.809	***	0.68	***	0.057	0.274	0.023	0.664
	CCR8	0.720	***	0.59	***	0.143	***	0.075	0.165
	STAT5B	0.230	***	0.14	***	−0.204	***	−0.177	***
	TGFβ(TGFB1)	0.472	0	0.31	***	0.006	0.901	−0.111	***
TAM	*CCL2*	0.624	0	0.45	***	0.205	***	0.126	***
	CD68	0.516	0	0.33	***	0.179	***	0.105	***
	IL10	0.610	***	0.43	***	0.117	***	0.012	0.817
T cell(general)	*CD3D*	0.932	***	0.88	***	0.172	***	0.251	***
	CD3E	0.938	0	0.89	***	0.263	***	0.176	***
	CD2	0.923	0	0.87	***	0.293	***	0.214	***
B cell	*CD19*	0.683	***	0.56	***	0.112	***	0.040	0.46
	CD79A	0.749	0	0.63	***	0.106	***	−0.003	0.96
Monocyte	*CD86*	0.806	0	0.68	***	0.210	***	0.122	***
	CD115(CSF1R)	0.738	***	0.60	***	0.241	***	0.145	***
M1 Macrophage	*INOS(NOS2)*	0.030	0.519	0.03	0.511	−0.003	0.95	−0.012	0.82
	IRF5	0.654	0	0.45	***	0.031	0.55	0.033	0.54
	COX2(PTGS2)	0.014	0.706	−0.11	***	0.123	***	0.032	0.56
M2 Macrophage	*CD163*	0.586	0	0.42	***	0.082	0.13	0.181	***
	VSIG4	0.607	0	0.47	***	0.233	***	0.156	***
	MS4A4A	0.670	0	0.51	***	0.248	***	0.166	***
Neutrophils	*CD66b(CEACAM8)*	−0.061	0.184	−0.04	0.461	−0.070	0.18	−0.098	***
	CD11b(ITGAM)	0.652	0	0.53	***	0.342	***	0.284	***
	CCR7	0.821	***	0.69	***	0.188	***	0.092	***
Dendritic cell	*HLA-DPB1*	0.857	0	0.75	***	0.269	***	0.191	***
	HLA-DQB1	0.792	0	0.66	***	0.246	***	0.168	***
	HLA-DRA	0.847	***	0.74	***	0.287	***	0.215	***
	HLA-DPA1	0.824	0	0.73	***	0.267	***	0.190	***
	BDCA−1(CD1C)	0.616	***	0.42	***	0.105	***	0.032	0.55
	CD11c(ITGAX)	0.636	***	0.43	***	0.234	***	0.163	***

SKCM, skin cutaneous melanoma; LIHC, liver hepatocellular carcinoma; Treg, regulatory T cell; TAM, tumor-associated macrophage; Th, T helper cell; Tfh, follicular helper T cell; Cor, *P* value of Spearman's correlation; None, correlation without adjustment. Purity and correlation adjusted by purity *$P < 0.01$; **$P < 0.001$; ***$P < 0.0001$.

Supplementary Table S11. Correlation constants and p-values in Figure 5 (TIMER v.1.0) ($p < 0.05$)

Gene markers		SKCM		LIHC	
		**Cor**	***p***	**Cor**	***p***
NK Cell	*KIR2DL3*	0.621	1.79×10^{-51}	−0.014	7.81×10^{-1}
	KIR3DL2	0.699	2.63×10^{-70}	0.046	3.82×10^{-1}
	KIR2DL4	0.737	7.41×10^{-82}	0.073	1.59×10^{-1}
	NCR1	0.599	3.39×10^{-47}	−0.002	9.73×10^{-1}
	NCR3	0.827	2.09×10^{-119}	0.218	2.32×10^{-5}

Supplementary Table S12. Correlation constants and p-values in Figure 7 (TIMER v.1.0) ($p < 0.05$)

Gene markers	SKCM		LIHC	
	**Cor**	***p***	**Cor**	***p***
GZMA	0.891	6.04×10^{-163}	0.271	1.16×10^{-7}
GZMB	0.869	0E + 00	0.029	5.8×10^{-1}
PRF1	0.871	1.01×10^{-46}	0.095	6.77×10^{-2}

Supplementary Figure S13. Correlations between *IL32* expression and chemokines (CXCL 9,10,11) in SKCM by GEPIA2. ($p < 0.05$)

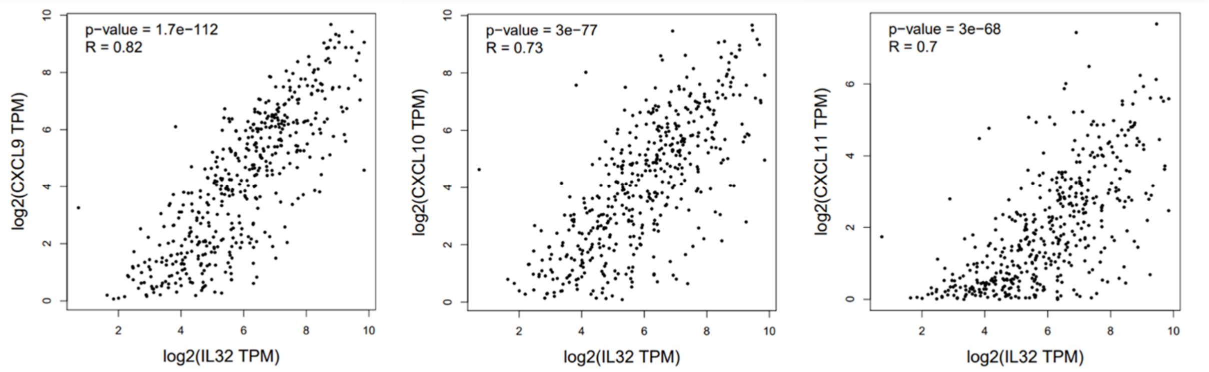

Supplement: Supplementary file 1 [file jcm-10-04691-s001.zip › JCM-1365860_Supplement_3rd revision.pdf]
